# The Swedish Hip Arthroplasty Register (www.shpr.se)

**DOI:** 10.3109/17453671003635918

**Published:** 2010-03-31

**Authors:** Johan Kärrholm

**Affiliations:** Department of Orthopaedics, Sahlgrenska University Hospital, MölndalSweden

## Introduction

In 1975, Peter Herberts initiated a national study of all reoperations after total hip replacement (THR) in Sweden. This study was designed as a trial lasting for almost 2 years (1976–1977). The goal was to learn more about severe complications of THR and thereby improve on the results. It was considered impossible to collect all primary hip replacements because of the vast number of operations.

The pilot study was designed as a retrospective one to evaluate whether a number of key parameters associated with reoperation of total hip arthroplasties could be collected for statistical analysis at a national level. Importantly, it was decided that any further operation after the primary procedure, regardless of whether the implants were exchanged (revision) or not, should be used as failure parameter. Later on, it turned out that the choice of data collected was well suited to analysis of outcome, which contributed to the future success of the Register, not least by stimulation of continuous learning and improvement.

Almost all orthopedics departments performing hip replacements participated in this project, which eventually comprised 513 reoperations. One important experience was that complications were far more common after reoperations than after primary surgery. About a third of all reoperations were associated with further complications (Ahnfelt et al. 1980).

Encouraged by the success of this pilot study, the orthopedics profession in Sweden was again asked if the members would participate in a prospective and continuous national multicenter study of reoperations after THR. At the initiation of this National Register in January 1979, all (at that time 62) but 1 department, which joined somewhat later, accepted to participate. Peter Herberts, who initiated the pilot study, became the leader of the Register and continued in this capacity for 30 years.

For the first 7 years, the Register was funded as a research project within an academic program. A research fellow, Lennart Ahnfelt, originally recruited for the pilot project, presented his thesis on this subject in 1986, which summarized the results up to 1983 (Ahnfelt et al. 1990). The project was presented internationally for the first time at the SICOT conference in London in 1984.

From the beginning, all individual reoperations were identified using the patient’s social security number. Medical records on every reoperation were collected. More than 100 parameters including demographic data, details of surgical technique, the implant used, the operating unit, and the type and history of the previously used implant or implants were recorded by specially trained secretaries. To enable calculation of implant survival, each hospital delivered information about implants used from 1967 onwards. From 1979 and until 1991, the hospitals continued to deliver details about primary hip arthroplasties on an aggregated level and for each year. This meant that it was not possible to track each individual primary operation to a social security number, which resulted in the need for approximations in order to calculate implant survival. This could, however, be done by the use of information from other governmental registers and statistically-based models (Herberts et al. 1989, Herberts and Malchau 1997). In a thorough evaluation, it turned out that the results from the first period (1979–1991) were valid (Söderman et al. 2000). From 1992, all primary total hip arthroplasties were also recorded in detail in the Register. This meant that each surgical procedure could be associated more firmly with patient demographics, the type of incision, a specific implant, and the technique of fixation.

In 1999, the recording was extended further by including more details of the implants. Information about, for example, sizes, offset, and implant materials became available. Despite the fact that this increasing amount of data collection over the years also meant that each clinic had to use more resources related to data collection, the register continued to have almost complete coverage.

Several factors were responsible for this success. One was the central secretarial unit that provided on-line support and recurring courses for the local secretaries, in addition to well-organized data collection and statistical support. Another key factor was continuous feedback of results to the profession.

Initially, only reoperations and implant survival with respect to design and method of fixation were reported at the national level. During the later part of the 1980s, each participating unit also gained access to their own frequency of, and reasons for, reoperations related to the national average. Even though this information could be both encouraging and troublesome for the individual hospitals, the potential power of it to allow monitoring and improvement of outcome was understood, appreciated, and practiced.

Much of the development of the Register was pioneering work. At an early stage, the Register also employed its own IT manager, Roger Salomonsson, who has been responsible for the development up until now. He and Henrik Malchau have been of great importance for analysis and publication of results—not least during the end of the last century, when information technology underwent such rapid development. The Hip Register was first in Sweden with on-line recording of primary THRs, as early as in 1999, and the technique now embraces all (at that time 78) but 1 participating unit. The importance of being in charge of your own IT development resulted in well-designed applications that were rapidly implemented.

In 2002, Göran Garellick introduced patient-reported outcome measurements (PROMs) to the register (Malchau et al. 2005). Patients scheduled for total hip arthroplasty filled in the EQ-5D form as a screening of health-related quality of life (HRQoL). In addition, pain and overall satisfaction were indicated on a VAS scale. In 2008, 77 of 79 units participated in this part of the registration.

At the turn of the century, there was increasing external demand—not least from journalists—to gain access to the results of THR at the departmental level. In a law suit, it was decided that no such data should be generally available. Even so, in 1999—in order to meet this demand and to play down the impact of this information—the Register holders decided to present some of the results openly, in cooperation with the orthopedic profession. Later on, several outcome parameters were added. This open access to key variables (to account for outcome) became the state of the art for Registers in Sweden. This is now supported by the Swedish National Board of Health and Welfare and the Swedish Association of Local Authorities and Regions (SALAR).

Today, the annual register report includes not only implant survival and the PROM variables. Reoperations within 2 years are used as a fast indicator of surgical quality. In addition, re-admission within 30 days and mortality within 90 days are reported for every operating unit. A simple case-mix factor based on sex, diagnosis, and age at surgery is used to indicate the average risk factor for the population operated at the individual hospitals. This is essential for comparisons between units, since the hospitals operate upon vastly different types of patients and have different commissions.

In 2005, a further step forward was taken when all hemiarthroplasties were recorded. Use of the same organization as that already being employed for total hips resulted in an immediate nationwide coverage. Even though these two registers are closely connected, the hemiarthroplasty register is a separate entity from the total hip arthroplasty register, with Cecilia Rogmark as project leader.

The annual report contains a general part covering demographic data, frequencies of procedures, choice of implant and fixation, and survival of the different implants or surgical techniques on a national basis. The same data are also reported regionally and to the individual units, but no single-surgeon data are recorded. To achieve full compliance, all surgeons have been assured of confidentiality. Each unit has full access to its own data, however, which allows a learning process at the local level.

Each year, one or several in-depth analyses are performed. Such analyses may involve influence of antibiotic prophylaxis, surgical approach, choice of implant design, or other issues. The idea is to provide feedback to the community to facilitate continuous improvement and provide motivation. This way of achieving high-quality hip replacement surgery as reflected by a low revision rate has obviously been successful. Over the years, the revision rate in Sweden has been decreasing continuously.

The huge amount of data in this national register is an excellent basis for epidemiological studies of factors that may have a possible influence on the outcome. By synchronization with other national databases, a number of factors related to healthcare accessibility, socioeconomic background, patient demography, associated illness, medication, and many other factors that may influence the outcome, can now be analyzed. Output from the register has often raised further questions and hypotheses, and has prompted further research.

The pioneering work surrounding the 2 orthopedics registries, the knee and hip arthroplasty registers, started as less well-known academic activities with only research funding and was sometimes questioned. It has now received both national and international recognition. As a result of the resulting continuous supply of data for national observational studies in Sweden and stimulation of a wide range of research projects, the potential of these registries has become more and more evident. In 2 decades, about 70 similar registries have developed covering much of Swedish healthcare. They all now receive government support and are used to provide guidelines for high-quality, evidence-based medical treatment.

## References

[1] Ahnfelt L

[2] Ahnfelt L, Andersson G, Herberts P (1980). Re-operation of total hip arthroplasties. Läkartidningen.

[3] Ahnfelt L, Herberts P, Malchau H, Andersson GB (1990). Prognosis of total hip replacement. A Swedish multicenter study of 4,664 revisions. Acta Orthop Scand (Suppl 238).

[4] Herberts P, Malchau H (1997). How outcome studies have changed total hip arthroplasty practices in Sweden. Clin Orthop.

[5] Herbert P, Ahnfelt L, Malchau H, Strömberg C, Andersson GBJ (1989). Presidential guest speaker. Multicenter clinical trials and their value in assessing total hip arthroplasty. Clin Orthop.

[6] Malchau H, Garellick G, Eisler T, Kärrholm J, Herberts P (2005). Presidential guest speaker: the Swedish Hip Registry: Increasing the sensitivity by patient outcome data. Clin Orthop.

[7] Söderman P, Malchau H, Herberts P, Johnell O (2000). Are the findings in the Swedish National Total Hip Arthroplasty Register valid? A comparison between the Swedish THR Register, the National Discharge Register and the National Death Register. J Arthroplasty.

